# Metabolomics Network Characterization of Resuscitation after Normocapnic Hypoxia in a Newborn Piglet Model Supports the Hypothesis That Room Air Is Better

**DOI:** 10.1155/2014/731620

**Published:** 2014-02-18

**Authors:** V. Fanos, A. Noto, T. Xanthos, M. Lussu, F. Murgia, L. Barberini, G. Finco, E. d'Aloja, A. Papalois, N. Iacovidou, L. Atzori

**Affiliations:** ^1^Neonatal Intensive Care Unit, Puericulture Institute and Neonatal Section, University of Cagliari, 09042 Cagliari, Italy; ^2^National and Kapodistrian University of Athens, Medical School, 10679 Athens, Greece; ^3^Department of Biomedical Sciences, University of Cagliari, 09042 Cagliari, Italy; ^4^Department of Neurological Sciences, University of Cagliari, 09042 Cagliari, Italy; ^5^Department of Anesthesiology, University of Cagliari, 09042 Cagliari, Italy; ^6^Forensic Science Department, Cagliari Teaching Hospital and University, University of Cagliari, 09042 Cagliari, Italy; ^7^Department of Obstetrics and Gynecology, Neonatal Division, National and Kapodistrian University of Athens, Medical School, 10679 Athens, Greece

## Abstract

Perinatal asphyxia is attributed to hypoxia and/or ischemia around the time of birth and may lead to multiorgan dysfunction. Aim of this research article is to investigate whether different metabolomic profiles occurred according to oxygen concentration administered at resuscitation. In order to perform the experiment, forty newborn piglets were subjected to normocapnic hypoxia and reoxygenation and were randomly allocated in 4 groups resuscitated with different oxygen concentrations, 18%, 21%, 40%, and 100%, respectively. Urine metabolic profiles at baseline and at hypoxia were analysed by ^1^H-NMR spectroscopy and metabolites were also identified by multivariate statistical analysis. Metabolic pathways associations were also built up by ingenuity pathway analysis (IPA). Bioinformatics analysis of metabolites characterized the effect of metabolism in the 4 groups; it showed that the 21% of oxygen is the most “physiological” and appropriate concentration to be used for resuscitation. Our data indicate that resuscitation with 21% of oxygen seems to be optimal in terms of survival, rapidity of resuscitation, and metabolic profile in the present animal model. These findings need to be confirmed with metabolomics in human and, if so, the knowledge of the perinatal asphyxia condition may significantly improve.

## 1. Introduction

Perinatal asphyxia is one of the leading causes of morbidity and mortality in the neonatal period. Worldwide, 4 million neonates annually suffer from birth asphyxia and 1 million die as a consequence of the condition. Among survivors, long term morbidity occurs, with brain disability being the most significant; at present though, the severity and outcome of this condition cannot be predicted [1, 2]. It has been reported that hypoxia and ischemia can cause damage not only to the central nervous system (28%) but also to various other organs such as the kidneys (50%), cardiovascular system (25%), and lungs (23%) [3], leading to multiorgan dysfunction, as blood redistribution to vital organs compromises renal, gastrointestinal, and skin perfusion, which in turn may result in large changes in the circulating metabolome [4]. The clinical diagnosis is based on several criteria, two of the main ones are being the evidence of cardiorespiratory/neurological depression and the evidence of acute hypoxic compromise with acidemia [5]. There is an on-going debate in the literature concerning the correct oxygen concentration to be used during neonatal resuscitation and this is attributed to gaps in knowledge in the field [6]. The latest guidelines for newborn resuscitation published in 2010, call for use of 21% oxygen, especially in term newborns, but there are still a number of unanswered questions [7].

Saugstad et al. [8] recently suggested the need for reviewing the use of oxygen as therapy for newborn resuscitation [6, 8]. Their animal studies clearly demonstrated that the use of room air may be better as it causes less damage by free radicals produced by higher oxygen concentration [9, 10]. In addition, it is well known that the hyperoxia that follows a hypoxic condition generates a condition of oxidative stress and multiorgan damage [11]. This is the reason why the current guidelines suggest titration of the oxygen administered during resuscitation.

In this context, metabolomics, the youngest “omics” technology, aims to integrate different levels of information for global understanding of biological systems. Metabolomics consists of holistic, rather than reductionist approach, on the molecules (such as genes, transcripts, proteins, and metabolites) that make up a cell, tissue, or organism [12]. The metabolomic analysis of biofluids or tissues has been successfully used in the fields of physiology, functional genomics, pharmacology, toxicology, and nutrition, demonstrating the ability of the method to discriminate different groups, due to the typical metabolic profile within each group [13–15]. Endogenous metabolites found in biofluids could describe the cellular phenotype. Moreover, through the rapid characterization of small molecules (metabolites), this new “omics” has the opportunity to explore the interactions such as genotype-phenotype and genotype-environment type, which means it is possible to have a snapshot of the metabolic status. In this context, the aim of metabolomics is to improve the early diagnosis, classification, and prediction of the evolution of a pathological condition. Metabolomics applications and techniques are in an exponential growth phase and it is already clear that this strategy will have a significant impact on the discovery of clinical and pharmacological biomarkers [16]. Significant work has been done over the last years, in this field during the perinatal period. Metabolomics has been shown to be a tool for the pharmacological treatments of patent ductus arteriosus (PDA), for the recognition of the newborn born with the asymptomatic cytomegalovirus infection and for the research of future biomarkers for conditions such as intrauterine growth restriction [17–19].

The aim of the study was to investigate whether different metabolomic profiles occurred according to oxygen concentration administered at resuscitation. We tested the hypothesis that the constitutive metabolic profile may be affected by the response to oxygen therapy and that different oxygen concentrations may induce discriminating changes in the metabolic pathways.

## 2. Material and Methods

The experimental protocol has recently been described by our group [20]. In brief, 40 male Landrace/large white newborn piglets, weighing 2.3−3.8 kg, were the subjects of the present study. Animals were sedated with an intramuscular injection of ketamine 10 mg/kg (Narketan, Vétoquinol UK Ltd.) and midazolam 0.5 mg/kg (Dormicum, Hoffmann-La Roche, Germany). Venous access was established via the marginal auricular vein and anesthesia was induced by administration of propofol 1 mg/kg (Diprivan, AstraZeneca) and fentanyl 10 *μ*g/kg (Fentanyl, Janssen-Cilag). Animals were then intubated. Normal saline 0.9% 10 mL/kg/h and 5 mL/kg/h of dextrose in water 5% were infused to prevent dehydration and hypoglycemia. Heart rate (HR), electrocardiogram (ECG), saturation of oxygen by pulse oximeter (SpO_2_), and rectal temperature (Matron, BPM 1000, VET, ET Medical Devices Spa) were monitored non-invasively. Body temperature was maintained at 38 ± 1°C with a table heating pad and an overhead heating lamp. An intravenous bolus of fentanyl 20 *μ*g/kg and cisatracurium 0.2 mg/kg (Nimbex, Abbott) were administered, after which they were mechanically ventilated (Soxil, Soxitronic, Felino, Italy). Ventilatory settings are tidal volume 10–15 mL/kg, pressure 19 cm H_2_O, and respiratory rate 30–40 breaths/minute aiming at end-tidal CO_2_ (ETCO_2_) of 35–45 mmHg. The fraction of inspired oxygen (fiO_2_) was adjusted between 0.21 and 0.25 in order to maintain target SpO_2_ 90–95%. Infusion of 8–10 mg/kg/h propofol and boluses of 10 *μ*g/kg fentanyl and 0.15 mg/kg cis-atracurium maintained anesthesia.

The right internal jugular vein and carotid artery were catheterized, via a paratracheal incision, with single-lumen catheters (S1UVC5.0, NeoCare; Klein-Baker Medical Co., San Antonio, TX, USA) which were connected to external transducers (Transpac, Abbott Critical Care Systems, USA), for continuous monitoring of central venous pressure, systolic, and mean and diastolic pressure of the carotid artery. The animals were stabilized for 30 minutes prior to experimentation.

The inspired fiO_2_ was then decreased to 0.06–0.08 to induce hypoxia, while the animals were maintained on the same settings of ventilation. Monitoring aimed at detecting either bradycardia (HR < 60 beats per minute) or severe hypotension (MAP < 15 mmHg) was performed. As soon as hemodynamic compromise occurred, hypoxemia (pO_2_ 30–50 mmHg) was confirmed on arterial blood gases and resuscitation began according to the newborn life support (NLS) algorithm [21]. Animals were allocated in 4 groups and were resuscitated with different O_2_ concentrations (18%, 21%, 40%, and 100%), until HR and MAP returned to 90% of baseline levels. When hemodynamic parameters returned to baseline values, the animals remained ventilated under anesthesia for 30 further minutes. Persisting asystole, despite 10 minutes of cardiopulmonary resuscitation, or return of the hemodynamic parameters to baseline values was the endpoints of the experiment. Surviving animals were humanely euthanatized by slow intravenous infusion of 30 mg/kg sodium thiopental (Pentothal, Hospira Enterprises BV, The Netherlands). Necropsy followed for examination of possible injury or abnormality.

Urine samples were collected from each animal at differend time points; A baseline urine sample was obtained before inducing the hypoxia and a second sample was collected once the animals were reoxygenated and stabilized for 30 minutes. In order to avoid the growth of bacteria in urine, 10 *μ*L of sodium azide solution (1% of NaN_3_ in H_2_O) was added in each sample.

### 2.1. Sample Preparation for ^1^H-NMR

Urine samples were centrifuged to remove insoluble material and stored at −80°C. Urine was prepared after being thawed at room temperature and centrifuged at 10,000 × rpm for 10 min at 4°C. 400 *μ*L of urine was added to a solution of 200 *μ*L of buffer solution pH 7.4 and 50 *μ*L of TSP (trimethylsilyl propanoic acid) in D_2_O 10 mM. The mixture was then dispensed into a 5 mm glass NMR tube (New Era, USA).

### 2.2. ^1^H-NMR Experiments

All ^1^H NMR spectra were carried out on a Varian UNITY INOVA 400 spectrometer. All samples were submitted to identical standard acquisition parameters and pulse. The sequence used was TNNOESY with mixing time of 0,150 seconds, a sat-delay of 2 sec, and a sat-power of 2 dB (decibels). Spectra were recorded at 300 K with a spectral width of 6000 Hz, a 90° pulse, an acquisition time of 2 s, a relaxation delay of 2 s, and 128 scans. The residual water signal was suppressed by applying a presaturation technique with low power radiofrequency irradiation for 2 s during relaxation. The total acquisition time was of 8 min. Chemical shifts were referred to the TSP single resonance at 0.00 ppm.

### 2.3. Statistical Analysis

The ^1^H-NMR spectra were divided in spectral domains of 0.04 ppm (bins) by selecting the regions 9.60–5.12 and 4.68–0.40 ppm. The spectral region between 4.68 and 5.12 ppm was eliminated to remove the effect of the residual water resonance. Bucketing was performed by MestReNova. The integrated area within each bin was normalized to a constant sum of 100 for each spectrum in order to minimize the effects of variable concentration among different samples. The final data set consisted of 208 variables (i.e., bins). The spectral data were imported into SIMCA-P+ program (Version 11.0, Umetrics, Umeå, Sweden), and Pareto was scaled before multivariate statistical analysis. The Pareto algorithm calculates for each variable the standard deviation *s*_*k*_ (standard deviation of the *k* variable), and the scaling weight is obtained as the inverse of standard deviation root $W=1/\sqrt{s_{k}}$. Each column is multiplied by the *W* factor and this gives the possibility of downweighting irrelevant and or noisy variables. Subsequently, the unsupervised principal components analysis (PCA) and a supervised partial least square discriminant analysis (PLS-DA) were applied. The results were graphically reported in the score plot and in the loading plot, where samples and variables, respectively, are projected in the multivariate space. In addition, the validity of the PLS-DA model was assessed by statistical parameters: the correlation coefficient *R* ^2^ and the cross-validation correlation coefficient *Q* ^2^. *R* ^2^ represents the goodness of the fit to the model and *Q* ^2^ reveals the predictability of the model [22]. Statistical differences in the resuscitation time were evaluated using Student's *t*-test with the Bonferroni correction.

## 3. Metabolites Identification

### 3.1. HMDB

The identification of the variable influence on the projection (VIP) score was performed using the Human Metabolome Database, (http://www.hmdb.ca/), which is an electronic database comprehending information about small molecule metabolites found in the human fluids [23–25]. The database contains more than 40 thousands metabolites as well as their abundance in the different human fluids.

### 3.2. Chenomx

The discriminating metabolites were quantified using the software Chenomx (Chenomx Inc., Edmonton, Canada). The software allows both the identification and quantification of the metabolites through an internal library of NMR signals of individual metabolites.

### 3.3. Ingenuity Pathways Analysis

The study of metabolic pathways was performed by using IPA (Ingenuity Systems, http://www.ingenuity.com/). IPA is a program in which all relevant information of the biomedical scientific literature is collected and coded. IPA allows exploring the value of the assumed correlation between the information generated by the analysis. The classification of this information takes into account, for example, the type of disease; the type of system function; and the type of molecule endogenous or exogenous, allowing identification of the connections between the different canonical pathways.

## 4. Results

**Figure 1:**
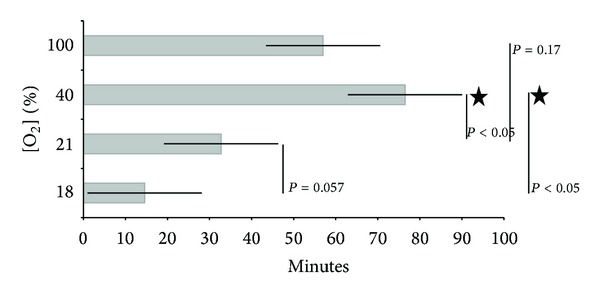
Resuscitation time (minutes) in the 4 groups of animals according to the oxygen concentration used for resuscitation (18%, 21%, 40%, and 100%).

In the 18% group, 5 of 10 experienced asystolic cardiac arrest episodes and 3 of them died (30% mortality rate). In the 21% group, 2 of 10 underwent asystolic cardiac arrest and one died (10%). In the 40% group 3 of 10 had asystolic cardiac arrest episodes with 2 deaths (20%). Finally, in the 100% group 3 of 10 showed asystolic cardiac arrest episodes and none survived (30%). Time to resuscitation is shown in Figure 1.

**Figure 2:**
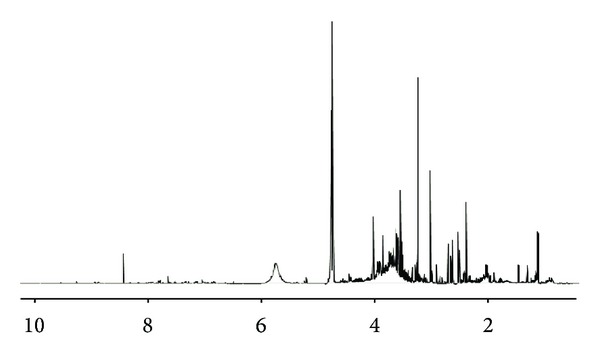
A 400 ^1^H-MHz NMR spectrum including aliphatic (1–4.5 ppm) and aromatic regions (6–8.5 ppm) is shown.

**Figure 3:**
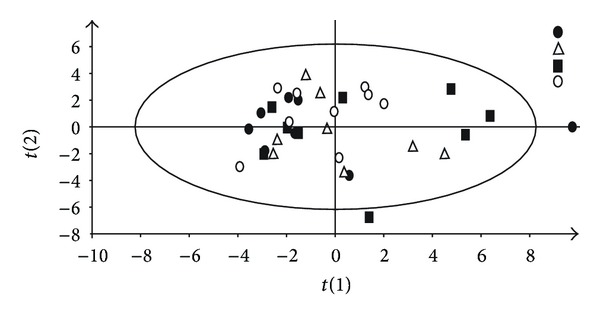
Principal component analysis (PCA) of urine at baseline withdrawal (before causing the experimental asphyxia); black circle: group that will be resuscitated at 18% (after experimental asphyxia), triangle: group that will be resuscitated at 21%, black square: group that will be resuscitated at 40%, and open circle: group that will be resuscitated at 100%.

Urine samples were prepared to perform ^1^H-NMR analysis. A representative urine NMR spectrum is shown in Figure 2. After processing, the spectra the resulting variables were analysed by using a multivariate statistical approach. The principal component analysis of the baseline sample did not show any cluster, indicating the homogeneity of the mathematical model. Two of the forty collected samples were not taken into consideration due to the fact that they were outside the confidence limit of T2 Hotelling distribution for scores, as shown in Figure 3.

**Figure 4:**
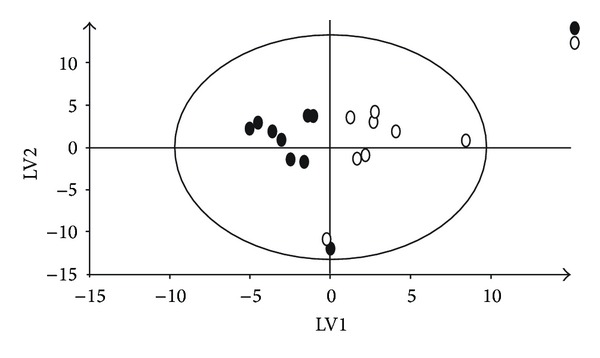
PLS-DA between the groups of baseline and reoxygenation at 21% of O_2_. The goodness of the matematical model was estimated as explained variance: *R* ^2^ *X* = 0.708 and *R* ^2^ *Y* = 0.641, and the predictive capability, *Q* ^2^, was 0.254.

**Figure 5:**
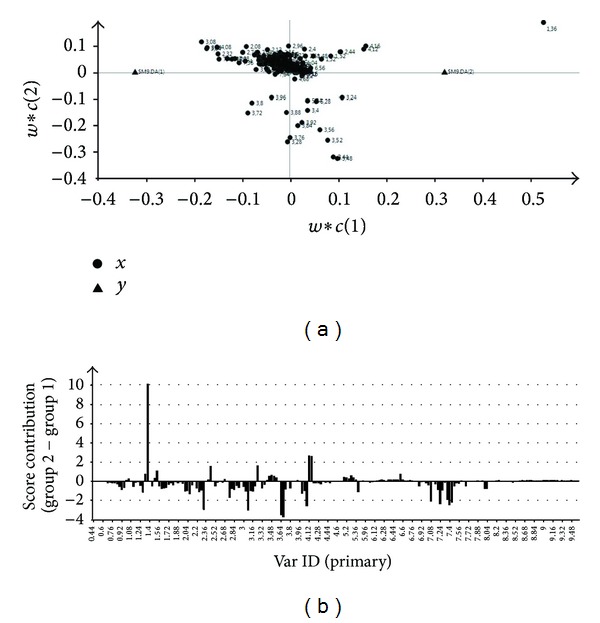
The loading (a) and contribution plots (b) of the metabolites between the baseline and the reoxygenation urine using 21% of O_2_ are shown.

**Figure 6:**
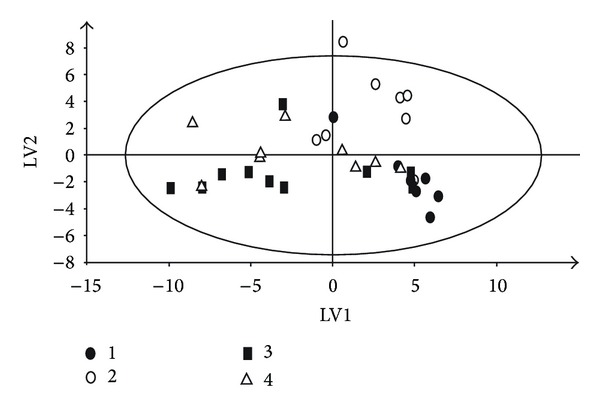
PLS-DA of the reoxygenation urine samples. Black circle: O_2_ at 18%, triangle: O_2_ at 21%, black square: O_2_ at 40%, and open circle: O_2_ at 100%.

Subsequently, analyses were performed for the 2 samples collected from each animal at (1) baseline and (2) reoxygenation, respectively. A PLS-DA model was able to highlight latent variables present within models. The 21% oxygen group (Figure 4) as well as 18%, 40%, and 100% oxygen groups showed clear separation in the metabolite of the urine of animals at baseline and reoxygenation. The compounds of importance were identified by inserting the list of individual peaks from the loadings plot (Figures 5(a) and 5(b)) in HMDB Database and using the recognition routine to match the peaks with substances NMR signals. All groups at the PLS-DA showed distinct separation (Figure 6).

By analysis with HMDB, the discriminant metabolites of the 4 groups were identified. Metabolic modifications were observed in the different groups when comparing the metabolic profile at time 0 and at the end of the experiment.

In addition to this, a bioinformatic analysis by means of IPA was performed excluding the metabolites that are common in all groups in order to highlight any differences as a function of oxygen concentration to which the individual groups were exposed. The software, beyond the discriminating metabolites generated by the supervised mathematical models, independently associates a number of metabolites that are related to the networks built by the model. The networks so resulting showed a peculiar effect on metabolism related to the different oxygen concentrations. Oxygen at 18% was associated with carbohydrate metabolism and at 21% with cellular function maintenance while at 40% and at 100% with free radical scavenging processes (see Table 1).

As an example, the metabolites variation between the two samples—baseline and resuscitation—associated with 21% of oxygen procedure is reported in Table 2.

## 5. Discussion

In the present study, we performed an ^1^H-NMR based metabolomics analysis of urine of newborn piglets before hypoxia and after reoxygenation using 4 different oxygen concentrations. The model mimics the perinatal asphyxia events, which occurs in about 4 millions newborns worldwide. It is of interest that 1 of 4 milion newborn has neurological disabilities as consequence of the oxygen deprivation and the treatment is still a matter of debate. It is well known that endogenous and/or exogenous stimuli such as oxygen administration are responsible for biological reactions inducing the production of a set of metabolites reflecting the metabolic status [26]. Although the use of oxygen at room air concentration is just advised for the beginning of the resuscitation process, it has been demonstrated that, for preterm and term birth asphyxia, the subsequent use of higher oxygen concentration may increase the formation of reactive species of oxygen.

Among these aspects of perinatal pathophysiology, the role of oxygen is still unclear. Oxygen is essential for the life of eukaryotic organisms, and yet quite paradoxically, a toxic substance. The toxicity of oxygen is linked to the ability to generate free radicals, a series of compounds containing at least one unpaired electron in the external orbital. The first targets are the phospholipids of biological membranes, but the action on DNA-damaging, cell structures, and mitochondrial has also been demonstrated [27].

The use of the ^1^H-NMR technique seems to be suitable for metabolomics experiment due to the fact that the analysis creates a wide spectrum consisting of several signals belonging to different classes of molecules. The piglet model of hypoxia-resuscitation appears to be a good model to extrapolate to human neonatal conditions. A metabolomic approach was previously used in asphyxiated newborn piglets [1, 10]. The use of 100% oxygen for resuscitation resulted in increased toxicity.

The major findings of the present study were that both the ^1^H-NMR coupled with multivariate and bioinformatics analyses pointed out that using oxygen at 21% seems to be better for the resuscitation in piglets with normocapnic hypoxia.

In fact, although the sample may be considered too small, a slight difference in the mortality rate has been observed, ranging from 10% for the 21% group up to 30% for the 18% and 100% O_2_ treated animals.

No inferences on the morbidity outcome (neurological status, MOFs, etc.) may be drawn by this experimental design, due to fact that this is an animal model and that all the animals have been promptly sacrificed in order to perform the histopathological analyses.

The proposed metabolomics analysis showed distinct metabolic profile characterizing each group. In fact, the analytical technique allows the simultaneous detection of hundreds of metabolites giving rise to a signature which is characteristic for each group, paving a new holistic way of looking at these molecules. In addition, the bioinformatic analysis of the urine metabolites characteristic of each group showed that resuscitation performed with lower oxygen concentration was associated with cellular homeostasis, cellular maintenance, and carbohydrates metabolisms. The metabolites responsible for the association were glucose, lactate, alanine, glyceric acid, pyruvic acid, malonic acid, glycine, succinate, 3-methyladenine, acetylglycine, glutaconic acid, 4-hydroxy-phenyl pyruvic acid, and 3-hydroxy-methyl glutarate. The group resuscitated with higher oxygen concentration was characterized by the presence of creatinine, urea, citric acid, tartaric acid, ethanol, glucose, and indoxyl sulfate. Indeed, the ^1^H-NMR metabolomic approach revealed changes in metabolites due to severe hypoxia.

A very recent paper by Skappak et al. [28] identified hypoxia in a newborn piglet model by urinary NMR metabolomic profile including, as relevant metabolites, lactate, alanine, pyruvic acid, and acetylglycine. Also, in a model of nonhuman primate, model of perinatal asphyxia analyzed by a metabolomic approach confirmed lactate, creatinine, malonic acid, and succinate as markers of asphyxia [29].

This study has several limitations: the relative paucity of the sample and the results obtained may not be directly translated into human scenario. The original aim of the experiment was to investigate the feasibility of the metabolomics approach to unravel the different outcomes, recently observed in other studies in the O_2_-level treatment (ranging from O_2_ percentage inferior to room air up to pure oxygen supplementation) of asphyxiated subjects.

Despite these limits—which need a further and ad-hoc experimental design—we believe that the therein proposed animal model gives an intriguing insight into the metabolic process occurring after the normocapnic hypoxia in response to the supplementation of different percentages of oxygen. The individual metabolomics responses of the forty animals which underwent normocapnic hypoxia are able to distinguish—with slight exceptions—the four treatment options (18, 21, 40, and 100%) and although no direct relationship may at the moment be drawn, it may suggest an O_2_-related metabolic response modulated by the oxygen availability.

## 6. Conclusion

The metabolomic approach here proposed clearly identifies four different metabolic signatures related to the different oxygen concentration treatments supported to the animals.

These differences underline the existence of complex metabolic pathways leading from the original hypoxic insult to the recovery/death outcome and they may also help to explaine the relative better result obtained with room air oxygen concentration.

These preliminary data support the hypothesis that the metabolomics approach could be a new tool for a more in-depth knowledge of the pathophysiological responses of the newborn to the resuscitation with different oxygen concentration.

## Figures and Tables

**Table 1:** Bioinformatic analysis of metabolites that characterized different oxygen concentration. Oxygen at 18% is associated with carbohydrates metabolism, at 21% with cellular function and maintenance, and at 40%–100% with free radical scavenging processes.

Oxygen concentration	IPA association	*P* value range
18%	Carbohydrate metabolism	5,43*E* − 10–4,28*E* − 3
21%	Cellular function and maintenance	3,05*E* − 21–7,9*E* − 10
40%	Free radical scavenging	2,84*E* − 14–1,21*E* − 14
100%	Free radical scavenging	1,62*E* − 12–4,91*E* − 4

**Table 2:** Discriminant metabolites as obtained by using HMDB and relevant metabolic changes compared to baseline are indicated for reoxygenation using 21% of O_2_.

Metabolites	Metabolic change after reoxygenation at 21% O_2_
Creatinine	↓
Sarcosine	↓
Glutamine	↓
Acetoacetate	↓
Phenylalanine	↓
Hippurate	↓
Trimethylamine	↓
Glucose	↑
Alanine	↑
Lactate	↑
3-Hydroxymethyl glutarate	↑
Succinate	↑
Malonic acid	↑
Glycine	—
Pyruvic acid	↓

## References

[1] Atzori L, Xanthos T, Barberini L (2010). A metabolomic approach in an experimental model of hypoxia-reoxygenation in newborn piglets: urine predicts outcome. *Journal of Maternal-Fetal and Neonatal Medicine*.

[2] Saugstad OD (2011). Reducing global neonatal mortality is possible. *Neonatology*.

[3] Laila HM, Mai AK, Nagy AE, Mohammed HZ, Raghdaa MA Multi-organ dysfunction in neonates with hypoxic-ischemic encephalopathy.

[4] Shah P, Riphagen S, Beyene J, Perlman M (2004). Multiorgan dysfunction in infants with post-asphyxial hypoxic-ischaemic encephalopathy. *Archives of Disease in Childhood*.

[5] Campbell DE, Imaizumi SO, Bernbaum JC (2008). *AAP Textbook of Pediatric Care*.

[6] Hansmann G (2004). Neonatal resuscitation on air: it is time to turn down the oxygen tanks?. *The Lancet*.

[7] Perlman JM, Wyllie J, Kattwinkel J (2010). Part 11: neonatal resuscitation: 2010 International Consensus on Cardiopulmonary Resuscitation and Emergency Cardiovascular Care Science with Treatment Recommendations. *Circulation*.

[8] Saugstad OD, Speer CP, Halliday HL (2011). Oxygen saturation in immature babies: revisited with updated recommendations. *Neonatology*.

[9] Liu J, Litt L, Segal MR, Kelly MJS, Yoshihara HAI, James TL (2011). Outcome-related metabolomic patterns from ^1^H/^31^ P NMR after mild hypothermia treatments of oxygen-glucose deprivation in a neonatal brain slice model of asphyxia. *Journal of Cerebral Blood Flow and Metabolism*.

[10] Solberg R, Enot D, Deigner H-P (2010). Metabolomic analyses of plasma reveals new insights into asphyxia and resuscitation in pigs. *PLoS ONE*.

[11] Vento M, Sastre J, Asensi MA, Viña J (2005). Room-air resuscitation causes less damage to heart and kidney than 100% oxygen. *American Journal of Respiratory and Critical Care Medicine*.

[12] Bollard ME, Stanley EG, Lindon JC, Nicholson JK, Holmes E (2005). NMR-based metabonomic approaches for evaluating physiological influences on biofluid composition. *NMR in Biomedicine*.

[13] Fiehn O (2002). Metabolomics—the link between genotypes and phenotypes. *Plant Molecular Biology*.

[14] Dunn WB, Broadhurst DI, Atherton HJ, Goodacre R, Griffin JL (2011). Systems level studies of mammalian metabolomes: the roles of mass spectrometry and nuclear magnetic resonance spectroscopy. *Chemical Society Reviews*.

[15] Atzori L, Antonucci R, Barberini L, Griffin JL, Fanos V (2009). Metabolomics: a new tool for the neonatologist. *Journal of Maternal-Fetal and Neonatal Medicine*.

[16] Atzori L, Griffin JL, Noto A, Fanos V (2012). Metabolomics: a new approach to drug delivery in perinatology. *Current Medicinal Chemistry*.

[17] Atzori L, Mussap M, Noto A (2011). Clinical metabolomics and urinary NGAL for the early prediction of chronic kidney disease in healthy adults born ELBW. *The Journal of Maternal-Fetal & Neonatal Medicine*.

[18] Atzori L, Antonucci R, Barberini L (2011). 1H NMR-based metabolomic analysis of urine from preterm and term neonates. *Frontiers in Bioscience*.

[19] Fanos V, Locci E, Noto A (2013). Urinary metabolomics in newborns infected by human cytomegalovirus: a preliminary investigation. *Early Human Development*.

[20] Aroni F, Xanthos T, Varsami M An experimental model of neonatal normocapnic hypoxia and resuscitation in Landrace/Large White piglets. *The Journal of Maternal-Fetal & Neonatal Medicine*.

[21] Nolan JP, Soar J, Zideman DA (2010). European resuscitation council guidelines for resuscitation 2010. *Resuscitation*.

[22] Want E, Masson P (2011). Processing and analysis of GC/LC-MS-based metabolomics data. *Methods in Molecular Biology*.

[23] Wishart DS, Jewison T, Guo AC (2013). HMDB 3.0—the human metabolome database in 2013. *Nucleic Acids Research*.

[24] Wishart DS, Knox C, Guo AC (2009). HMDB: a knowledgebase for the human metabolome. *Nucleic Acids Research*.

[25] Wishart DS, Tzur D, Knox C (2007). HMDB: the human metabolome database. *Nucleic Acids Research*.

[26] Barouxis D, Chalkias A, Syggelou A, Iacovidou N, Xanthos T (2012). Research in human resuscitation: what we learn from animals. *The Journal of Maternal-Fetal & Neonatal Medicine*.

[27] Valko M, Leibfritz D, Moncol J, Cronin MTD, Mazur M, Telser J (2007). Free radicals and antioxidants in normal physiological functions and human disease. *International Journal of Biochemistry and Cell Biology*.

[28] Skappak C, Regush S, Cheung PY, Afamko DJ (2013). Identifyng hypoxia in a newborn piglet model using NMR metabolomic profiling. *PLos ONE*.

[29] Beckstrom AC, Humston EM, Snyder LR, Synovec RE, Juul SE (2011). Application of comprehensive two-dimensional gas chromatography with time-of-flight mass spectrometry method to identify potential biomarkers of perinatal asphyxia in a non-human primate model. *Journal of Chromatography A*.

